# Prediction of Early Recurrence After Surgery for Liver Tumor (ERASL): An International Validation of the ERASL Risk Models

**DOI:** 10.1245/s10434-021-10235-3

**Published:** 2021-07-07

**Authors:** Berend R. Beumer, Kosei Takagi, Bastiaan Vervoort, Stefan Buettner, Yuzo Umeda, Takahito Yagi, Toshiyoshi Fujiwara, Ewout W. Steyerberg, Jan N. M. IJzermans

**Affiliations:** 1 Erasmus MC Transplant Institute Department of Surgery, Division of HPB & Transplant Surgery, University Medical Centre, Rotterdam, The Netherlands; 2 Department of Gastroenterological Surgery, Okayama University Graduate School of Medicine, Dentistry, and Pharmaceutical Sciences, Okayama University Hospital, Okayama, Japan; 3 Department of Biomedical Data Science, Leiden University Medical Centre, Leiden, The Netherlands

## Abstract

**Background:**

This study aimed to assess the performance of the pre- and postoperative early recurrence after surgery for liver tumor (ERASL) models at external validation. Prediction of early hepatocellular carcinoma (HCC) recurrence after resection is important for individualized surgical management. Recently, the preoperative (ERASL-pre) and postoperative (ERASL-post) risk models were proposed based on patients from Hong Kong. These models showed good performance although they have not been validated to date by an independent research group.

**Methods:**

This international cohort study included 279 patients from the Netherlands and 392 patients from Japan. The patients underwent first-time resection and showed a diagnosis of HCC on pathology. Performance was assessed according to discrimination (concordance [C] statistic) and calibration (correspondence between observed and predicted risk) with recalibration in a Weibull model.

**Results:**

The discriminatory power of both models was lower in the Netherlands than in Japan (C statistic, 0.57 [95% confidence interval {CI} 0.52–0.62] vs 0.69 [95% CI 0.65–0.73] for the ERASL-pre model and 0.62 [95% CI 0.57–0.67] vs 0.70 [95% CI 0.66–0.74] for the ERASL-post model), whereas their prognostic profiles were similar. The predictions of the ERASL models were systematically too optimistic for both cohorts. Recalibrated ERASL models improved local applicability for both cohorts.

**Conclusions:**

The discrimination of ERASL models was poorer for the Western patients than for the Japanese patients, who showed good performance. Recalibration of the models was performed, which improved the accuracy of predictions. However, in general, a model that explains the East–West difference or one tailored to Western patients still needs to be developed.

**Supplementary Information:**

The online version contains supplementary material available at 10.1245/s10434-021-10235-3.

Liver cancer is among the top five most commonly occurring malignancies, ranking as the fourth highest cause of cancer-related deaths worldwide.^1^ In this study, hepatocellular carcinoma (HCC) accounted for the vast majority of patients with primary liver cancer.

For patients with sufficient liver reserve, tumor resection has been shown to improve survival.^2^ Resection is aimed at cure, but for 30–50% of patients, the cancer recurs within the first 2 years.^3^,^4^ Therefore, early recurrences form a major challenge because survival in this group is substantially lower and the gain from the performed surgery is less clear. Preoperative risk prediction of HCC recurrence can aid patients and doctors in deciding whether to perform major surgery. Postoperative risk prediction may help in deciding adjuvant therapy and the intensity of follow-up treatment.

In 2018, Chan et al.^5^ published the pre- and postoperative early recurrence after surgery for liver tumor (ERASL) models using well-established clinical parameters to categorize patients into low-, intermediate-, and high-risk groups. The ERASL models are suggested as the first in the field able to provide personalized survival predictions.

A key step before use of any risk score in clinical practice is to validate the performance of the model.^6^,^7^ Ideally, the validation process should be performed on numerous independent samples, with assessment of whether the model is correctly specified, the extent to which it can discriminate between high- and low-risk patients, and whether the predicted survival probabilities match the observed data.

Chan et al.^5^ have assessed the discriminatory power and calibration of the ERASL models in external validation cohorts from four countries: Japan, the United States, China, and Italy. Although the authors used external cohorts for validation, the absence of an independent validation study is a major restriction for use of the ERASL models in daily practice. Moreover, the calibration was assessed only visually, and the analysis relied heavily on categorization of the patients into risk groups. It should be stressed that the model was derived in a hepatitis B-prevalent region, and it remains to be determined how well the model generalizes to other geographic areas where other causes of liver disease are more dominant. Therefore, we performed a fully independent validation using datasets of resected HCC patients from The Netherlands and Japan.^6^,^7^

## Patients and Methods

This retrospective cohort study is reported according to the critical appraisal and data extraction for systematic reviews of the prediction modelling studies (CHARMS) checklist and the transparent reporting of a multivariable prediction model for individual prognosis or diagnosis (TRIPOD) guidelines (Electronic supplementary Table 1).^8^,^9^

### Patients

Data were obtained from the Erasmus Medical Centre, Rotterdam, the Netherlands, and from the Okayama University Hospital, Okayama, Japan. The ethical requirements in both centers were approved (ID: MEC-2019-0498, MEC-2018-1544). The datasets contain the clinical parameters from patients with HCC who received first-time resection with curative intent.

The patients were referred either from other hospitals or from the program that routinely screened patients with chronic hepatitis B, chronic hepatitis C, or cirrhosis. In the Erasmus MC, the screening involved ultrasonography and measurement of alpha-fetoprotein (AFP), which could be combined with computed tomography (CT) or magnetic resonance imaging (MRI), every 9 to 12 months.

In Okayama, additional screening instruments included des-gamma-carboxy prothrombin (DCP) and the lectin-reactive fraction of AFP (AFP-L3). For the Okayama cohort, a default interval of 6 months was used, which was intensified to every 4 months for the patients with advanced cirrhosis.^10^

In both centers, patient eligibility for surgery was assessed at a multidisciplinary tumor board meeting based on performance status, liver function, and resectability of the tumor. Follow-up evaluation, including CT and laboratory assessment, generally was performed 3, 6, and 12 months after discharge and annually for a period of at least 5 years.

Recurrence-free survival (RFS), the dependent variable, was defined as the time between surgery and recurrence. Patients were censored at the date of their last radiologic examination if they had been lost to follow-up evaluation or had died without recurrence. In concordance with the derivation study, the follow-up evaluation was truncated 2 years after surgery.

The preoperative covariates used in the ERASL scores are gender, albumin (g/l), total bilirubin (*μ*mol/l), serum AFP (*μ*g/l), diameter of the largest tumor (cm), and number of tumors. Microvascular invasion (MVI) is the only covariate added in the postoperative risk score, defined as tumor invasion of vessels identified on histologic microscopic examination. Patients were excluded from analysis if they had one or more missing values of these covariates. The complete case analysis and definitions of covariates were in line with those of the derivation study.^5^ The full specification of the albumin-bilirubin (ALBI) grade and the ERASL scores is presented in Electronic supplement 2.

### Methods

The validation process consisted of three stages in which the misspecification, discrimination, and calibration were assessed using the methods and performance measures presented by Royston and Altman,^11^ Rahman et al.,^12^ van Houwelingen,^13^ and Steyerberg,^14^ and summarized in Steyerberg.^15^ In the following description, the linear predictor (LP) is the linear combination of the covariates and associated weights published by Chan et al.^5^ A patient’s risk score is the scalar value resulting from the evaluation of this patient’s LP.

### Model Validity

As an overall test to determine whether the relative risks were correctly specified, the calibration slope was computed. The measure was calculated by performing a Cox proportional hazard (CPH) regression with the LP as the only covariate. With this measure, a coefficient sufficiently close to 1 provides the first evidence that the model is correctly specified.^13^

Subsequently, we investigated the extent to which the coefficients of individual covariates would differ if they were re-estimated in the validation cohort. In these regressions, a CPH model was estimated in which all the individual covariates were added alongside the LP as an offset variable, with its coefficient constrained to 1. The coefficients represented the differences in hazard ratios between the derivation and validation cohorts. A likelihood ratio test was used to assess whether the estimated coefficients jointly were significantly different from zero.

### Discrimination

We evaluated the same performance metrics used by Chan et al.^5^ to aid the comparison, and included Harrell’s C-index, Gönen and Heller’s K, Royston and Sauerbrei’s Rd squared,^2^ and the time-dependent area under the receiver operating characteristic curve (tdAUC).

### Calibration

Two types of calibration plots to display the extent to which the predictions matched the observed data were used. First, the average predicted survival probabilities over the Kaplan Meier curve were superimposed per risk group.^11^ In a second plot, the predicted survival probabilities 1 and 2 years after surgery were plotted against the Kaplan-Meier estimates at these time points and compared against the 45° line. Both calibration plots heavily rely on arbitrarily formed risk groups and do not quantify the lack of fit. Hence, the ERASL models were embedded in a Weibull calibration model (Eq. 1). The parameter μ represents the accuracy of the overall risk level, with γ representing the impact of the LP and σ representing the shape of the baseline hazard. The variable T* represents the event time (t) transformed using the cumulative baseline function. It is assumed that the error term $W$ follows a type 1 extreme value distribution.^13^

1 $$ln (T*)=\upmu +\upgamma (LP)+\upsigma W$$

Thereafter, the Weibull model was used to achieve recalibrated survival probabilities using the following equation:

2 $$S{\left(t|LP\right)}_{cal}=P\left[T>t|LP\right]=exp\left(-exp\left(\frac{1}{\upsigma }\left(ln\left(-ln\left({S}_{0}\left(t\right)\right)\right)-\upmu -\gamma LP\right)\right)\right)$$

### Model Updating

We used forward selection, which starts with the CPH model using only the LP. Hereafter, in successive rounds, the covariate with the smallest *p* value was added to the model. To investigate the impact of hepatitis B and C infections, these were added one by one to the model, with the LP constraint to 1.

### Statistical Software

Data manipulations were performed in Python 3.7.^16^ Statistical analysis was performed in R version 3.5.1^17^ using the following packages: survival, rms, survAUC, survcomp, and boot.^18^–^22^ The R code with detailed comments is supplied in Electronic supplemental file 2.

## Results

### Patient Cohorts

The Rotterdam cohort comprised data from 312 patients collected from January 2000 through December 2017. Missing data included 25 albumin, 11 total bilirubin, 12 AFP, 3 tumor size, 3 tumor number, and 27 MVI values. For the validation of the ERASL-pre model, 33 patients (11%) were excluded due to missing data on at least one of these variables. For the validation of the ERASL-post model, the data for 53 patients (17%) were excluded. Ultimately, data for 279 and 259 patients were eligible to be analyzed for the ERASL-pre and ERASL-post models, respectively.

In Rotterdam, disease recurrence rate and survival status of the patients were last updated in February 2020. Recurrence was experienced by 164 of the 279 patients analyzed for the ERASL-pre model. For 116 of these patients, the recurrence developed 2 years after surgery. The median follow-up period was 5 years, with 77% the patients followed up for at least 2 years.^23^

Of the 259 patients analyzed for the ERASL-post model, 157 were found to have recurrence. For 110 of these patients, the recurrence developed during the first 2 years after surgery. The follow-up period for 78% of these patients was at least 2 years, with a median follow-period of 5 years.

The Okayama dataset comprised patient data collected between January 2007 and December 2017 for 392 patients. This dataset had no missing values. The disease recurrence rate and survival status were last updated in February 2020. A total of 196 patients had disease recurrence, with 139 of the patients experiencing recurrence in the first 2 years after surgery. The median follow-up period was 5 years, with 85% of the patients followed up for at least 2 years.

### Baseline Comparability

Baseline characteristics are summarized in Table 1. Information from the Hong Kong derivation cohort has been added to aid comparison. In the Okayama cohort, the cause of the HCC was most often ascribed to hepatitis C (47%), whereas in the Hong Kong cohort, hepatitis B (84%) was most prominent. In the Rotterdam cohort, hepatitis infections occurred less often, including hepatitis B in 25% and hepatitis C in 15% of the patients. In Rotterdam, the median tumor size was larger with 59 mm versus 35 mm in Okayama.

**Table 1:** Baseline characteristics of the Rotterdam, Okayama, and original derivation cohorts

Variables	Rotterdam *n* (%)	Okayama *n* (%)	Hongkong (derivation) *n* (%)
*N*	279	392	451
Male gender	192 (70)	311 (79)	387 (86)
Mean age (years)	60 ± 14	67 ± 10	56 ± 11
Hepatitis B	70 (25)	97 (27), n=356	380 (84)
Hepatitis C	41 (15)	185 (47)	18 (4)
Child-Pugh grade	(*n* = 274)		
A	262 (96)	383 (98)	442 (98)
B	12 (4)	9 (2)	9 (2)
C	0 (0)	0 (0)	0 (0)
ALBI grade			
1	225 (81)	257 (66)	329 (73)
2	51 (18)	134 (34)	119 (26)
3	3 (1)	1 (0)	3 (1)
Mean albumin (g/L)	42 ± 5.8	40 ± 4.6	40 ± 4.4
Median bilirubin: μmol/L (IQR)	10 (7–15)	12 (9–15)	10 (7–13)
Median AFP: μg/L (IQR)	9 (3–148)	10 (4–78)	52 (5–585)
Major resection	135 (48)	145 (37)	NA
Open RFA	22 (8)	3 (1)	NA
Positive margin	57 (22)	10 (3)	NA
Median tumor size: mm (IQR)	59 (32–96)	35 (23–60)	40 (25–60)
Solitary tumor	221 (79)	277 (71)	350 (77)
Microvascular invasion	150 (58) n=259	113 (29)	121 (27)
Recurrence	164 (56)	196 (50)	NA
Intrahepatic recurrence^a^	112 (68)	156 (80)	NA
Recurrence within 2 years	116 (42)	139 (35)	162 (35.9)
Median recurrence-free survival: months (95% CI)	25 (20–34)	48 (36–73)	66 (48–83)
Treatment of recurrence			
Re-operation	33 (20)	36 (18)	NA
Salvage liver transplantation	8 (5)	1 (1)	NA
Ablation	51 (31)	95 (48)	NA
TACE	12 (7)	117 (60)	NA
Radiotherapy/yttrium	21 (13)	23 (12)	NA
Chemotherapy	38 (23)	78 (40)	NA

ALBI grade, albumin bilirubin grade; IQR, interquartile range; AFP, alpha-fetoprotein; RFA, radiofrequency ablation; CI, confidence interval; TACE, transarterial chemoembolization

^a^Recurrent disease confined to the liver; the percentage is calculated with respect to the total number of recurrences.

Major resection and resection combined with radiofrequency ablation were both more common in the Rotterdam cohort (Electronic supplementary Table 2). Postoperatively, MVI differed as well, with a 58% rate for the Rotterdam cohort, a 29% rate for the Okayama cohort, and a 27% rate for the Hong Kong cohort (Table 1). Differences were found in the time until recurrence, including a median of 25 months in the Rotterdam cohort, 48 months in the Okayama cohort, and 66 in the Hong Kong cohort. This finding also was reflected in the baseline survival functions (Electronic supplementary Figure 1). In the Okayama cohort, recurrences were more often intrahepatic than in the Rotterdam cohort. Finally, treatment of HCC recurrence varied between centers. Most notable was the more frequent use of transarterial chemoembolization (TACE) (Okayama 60% vs Rotterdam 12%) and chemotherapy (Okayama 40% vs Rotterdam 23%) in the Okayama cohort.

In the Rotterdam cohort, the mean ERASL-pre and ERASL-post scores were 2.0 ± 0.70. In the Okayama cohort this value was 2.1 ± 0.88 for the ERASL-pre score and 1.9 ± 0.90 for ERASL-post score. The medians in the Hong Kong derivation cohort differed from the those published for the pre- and post-scores. Furthermore, for both the pre- and post-scores, the Rotterdam distributions were symmetric, whereas for the Hong Kong and Okayama cohorts were skewed to the right.

The ERASL-pre model assigned only four patients of the Rotterdam cohort to the high-risk group (Table 2). Furthermore, the differences between risk groups in terms of median survival and hazard ratios were greater overall in the Okayama cohort than in the Rotterdam cohort. In addition, the differences between the risk groups increased as information regarding the MVI was added in the ERASL-post score.

**Table 2:** Median the recurrence-free survival (RFS) rate and hazard ratio (HR) for each risk group^a^

Group	Rotterdam			Okayama		
	Low	Intermediate	High	Low	Intermediate	High
ERASL-pre						
*n* (%)	217 (78)	58 (21)	4 (1)	285 (73)	80 (20)	27 (7)
Median RFS: months (95% CI)	26 (21–39)	17 (9–43)	9 (5–NR)	100 (60–118)	14 (11–24)	4 (3–19)
HR (95% CI)	1	1.4 (0.9–2.2)	2.8 (0.9–8.9)	1	3.0 (2.1–4.3)	6.2 (3.7–10.4)
ERASL-post						
*n* (%)	163 (63)	93 (36)	3 (1)	286 (73)	80 (20)	26 (7)
Median RFS: months (95% CI)	33 (23; 41)	17 (11–26)	7 (0.49–NR)	99 (60–118)	12 (8–20)	4 (3–18)
HR (95% CI)	1	1.7 (1.1–2.4)	5.5 (1.7–17.6)	1	3.5 (2.4–5.0)	6.9 (4.1–11.8)

ERASL, early recurrence after surgery for liver tumor; CI, confidence interval; NR, not reached

^a^Number of patients per risk group, median survival, and the relative risk. The low-risk group was taken as the reference category.

### Model Validity

All discrimination measures were higher in the Okayama cohort than in the Rotterdam cohort. Furthermore, all discrimination measures were higher for the ERASL-post score than for the ERASL-pre score (Table 3). The ERASL-pre model attained a C-index of 0.57 (95% CI 0.51–0.63) in the Rotterdam cohort, whereas in the Okayama cohort, a C-index of 0.69 (95% CI 0.65–0.73) was found.

**Table 3:** Measures of discrimination

Measure of discrimination	ERASL-pre			ERASL-post	
	Rotterdam *n* (95% CI)	Okayama *n* (95% CI)		Rotterdam *n* (95% CI)	Okayama *n* (95% CI)
Harrell’s C-index	0.57 (0.51 to 0.63)	0.69 (0.65 to 0.73)		0.62 (0.56 to 0.68)	0.70 (0.66 to 0.74)
Gönen & Heller's K	0.67 (0.65 to 0.69)	0.7 (0.68 to 0.72)		0.67 (0.65 to 0.69)	0.70 (0.68 to 0.72)
Royston-Sauerbrei's Rd^2^	0.03 (− 0.03 to 0.09)	0.24 (0.12 to 0.36)		0.09 (0.01 to 0.17)	0.29 (0.17 to 0.41)
tdAUC	0.6 (0.48 to 0.72)	0.76 (0.74 to 0.78)		0.72 (0.7 to 0.74)	0.77 (0.75 to 0.79)

tdAUC, area under time-dependent receiver operating characteristic curve

Standard errors (SEs) were estimated from 200 bootstrap samples.

**Fig. 1:**
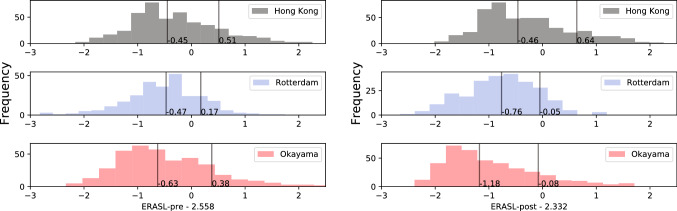
Distribution early recurrence after surgery for liver tumor (ERASL) risk scores. Distributions of the ERASL pre- and post-risk scores in the Hong Kong derivation cohort and in the Rotterdam and Okayama validation cohorts. The scores are centred on the median values described in the paper by Chan et al.^5^ In each histogram, the left black line represents the 50th percentile, and right black line represents the 85th percentile.

Significant differences in the prognostic effects were found for the both the ERASL-pre and ERASL-post models in the Rotterdam cohort, and for the ERASL-pre model in the Okayama cohort. The slope for the preoperative model in the Rotterdam cohort deviated the most, with a value of 0.32 (95% CI 0.04–0.59 (Electronic supplementary Table 3). Specifically, for both the Rotterdam and Okayama cohorts, the impact of gender was significantly smaller. Additionally, the impact of an ALBI grade greater than 1 was significantly smaller in the Rotterdam cohort (Electronic supplementary Table 4) (Fig. 1).

**Fig. 2:**
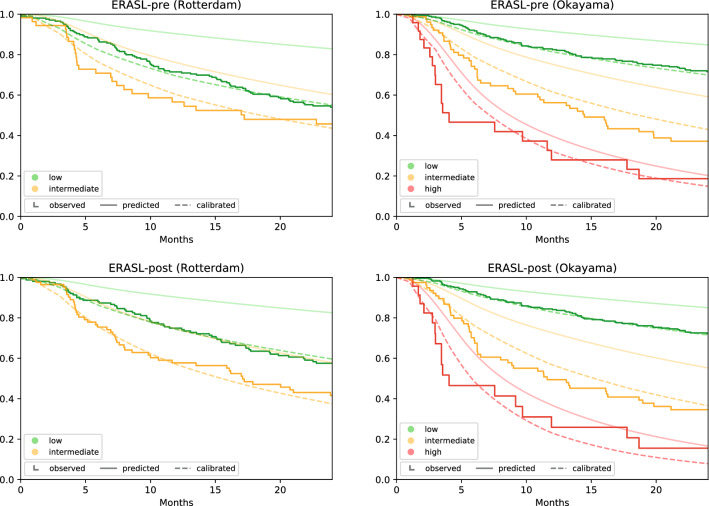
Calibration plot. The smooth solid lines represent the average predictions per risk group from the original model. The dashed curves represent the calibrated survival probabilities

The ERASL models systematically overestimated the RFS for the low- and intermediate-risk groups (Fig. 2). The results from the recalibration confirmed the mismatch in overall risk level, with µ coefficients ranging from − 2.21 to − 0.83 and all significantly different from zero (*p* < 0.001) (Electronic supplementary Table 5). Also, the exaggerated impact of prognostic factors in the LP was confirmed with gamma coefficients ranging from − 0.90 to − 0.39, all significant (*p* < 0.001). After use of these coefficients to recalibrate the model, the model matched the observed Kaplan-Meier curves much closer. The calibration plots confirmed that the re-calibration mainly corrected this optimism (Electronic supplementary Figure 2).

### Model Extension

Addition of hepatitis B and C infections to the LP did not achieve significance in either the pre- or postoperative models for either cohort (Electronic supplementary Table 6). Modification of risk score coefficients also was investigated. Starting with only the LP, variables were added in a forward selection manner. For the preoperative model in the Rotterdam cohort, ln(AFP) (0.08; 95% CI 0.02–0.14; *p* = 0.02) was significantly different from zero (Electronic supplementary Tables 7 and 8).

In the postoperative setting for the Rotterdam cohort, micro-vascular invasion (0.64; 95% CI 0.12–1.16; *p* = 0.02) and ln(AFP) (0.07; 95% CI 0.01–0.13; *p* = 0.03) were significant. For the Okayama cohort, the only variable achieving significance in the pre- and postoperative models was gender, with respective coefficients of − 0.67 (95% CI − 1.12 to − 0.22; *p* < 0.001) and − 0.62 (95% CI − 1.06 to − 0.18; *p* < 0.001).

## Discussion

This study assessed the validity of the ERASL models in independent cohorts to evaluate its applicability in daily practice. The ERASL models quantify the likelihood that a patient with HCC will experience early recurrence after resection. Preoperatively this information may help decision-making when the risk of major surgery should be balanced against the risk of early recurrence. Postoperatively, the model enables clinicians to provide the appropriate surveillance to detect recurrent HCC and additional treatment, including re-resection or salvage transplantation.^24^–^26^

The most important aspect of a model’s performance is its discriminatory power to separate low- and high-risk patients. Our evaluation of discriminatory power, as reported in Table 3, can best be viewed in relation to the results published by Chan et al.^5^ In the Rotterdam cohort, the model performed least (0.57), with a C-index similar to that for the Italian validation cohort (0.60) and substantially lower than for the Hong Kong derivation cohort (0.71). In the European cohorts, the discriminatory power of the risk score can be considered low (i.e., C-index ≤ 0.6). In contrast, the models in the Okayama cohort (C-index, 0.69) almost achieved the same level attained in the derivation dataset and discriminated well compared with other models.

Apart from a low discriminative performance in the Western cohort, we found that the original models were poorly calibrated for both the Western and Eastern cohorts. The high-risk group appeared to fit better, although the number of cases supporting the Kaplan-Meier curve in this group was minimal. Poor calibration caused the original ERASL models to exaggerate the difference in survival between risk groups and systematically overestimated the RFS. This systematic bias also was visible in all validation cohorts presented in the supplements of the derivation paper, confirming our results.^5^ Using the Weibull calibration model, for each cohort and model, we estimated three parameters to quantify and correct the calibration. However, we noted that the calibration parameters need to be validated in turn before wider adoption.

Another point of interest is that the published 50th and 85th quantiles on which the risk score thresholds are based, did not match the quantiles of the derivation cohort. The proportion of patients assigned to the intermediate- and high-risk groups were therefore smaller than the intended 35% and 15%. This also held for the other validation cohorts described in the derivation study.^5^ Therefore, the summary statistics describing the high-risk group are less stable and warrant a different interpretation because they describe even more extreme cases.

Regarding the prognostic profiles, the right skewness of the Japanese cohort matched that of the derivation cohort, whereas in the Rotterdam cohort a more symmetric distribution was observed. Consequently, in the Rotterdam cohort, fewer patients were assigned to the high-risk group than in the Okayama and Hong Kong cohorts. Interestingly, in the Rotterdam cohort, the risk of early recurrence was found to be the highest of all three cohorts. This mismatch between few high-risk predictions and high rates of early recurrence underscores that the models lack sensitivity and cannot be used in daily practice for Western patients.

A candidate risk factor that might explain this difference is the presence of hepatitis B or C. In the current study, the proportion of patients presenting with hepatitis B or C strongly differed between the cohorts. In both the offset regressions and the forward selection procedure, however, neither of these variables was significant. It therefore appears that although hepatitis B and C are important factors for diagnosis and treatment, they do not accurately reflect the severity of HCC after the other variables in the ERASL model have been taken into account.

To explore directions for further research, we re-estimated variables that have already been incorporated. For the Okayama cohort, we found that the coefficient for gender differed significantly from zero in both the pre- and postoperative settings using offset regression and the forward selection procedure. The suggested modification almost completely negated the effect of the gender covariate used in the ERASL models. This result confirms the concern raised earlier by Zhang et al.^27^ in their letter to the editor, in which they were surprised that gender was such a strong predictor. They performed a multi-center study in which they found similar rates of early recurrence between males and females (43.3% vs 42.0%; *p* = 0.728).

The misspecification tests for the Rotterdam cohort in the postoperative setting were less clear. Whereas gender ALBI grade and tumor size covariates were significant in the offset regression, the covariates for ln(AFP) and micro-vascular invasion were significant when the forward selection procedure was followed. In the latter, the changes in hazard ratio were substantial, with an additional 8% risk increase per unit of ln(AFP) and an additional 89% risk increase for MVI. It is remarkable that the higher impact of MVI in the Rotterdam cohort was paired with a high incidence.

High incidence of MVI also was found in the validation cohorts from the United States and Italy. Because the higher risk was paired with a higher incidence in Western cohorts, our results reflect differences in the timing of the diagnosis and underlying tumor biology between Eastern and Western cohorts rather than differences in definition.^28^ This hypothesis is further supported by the fact that the median tumor size in the Rotterdam cohort was almost double that in the Okayama cohort. In addition, early recurrences occurred more often in Rotterdam, and when recurrence was found, it was less often confined to the liver.

Although our research was not designed to inspect East–West differences, we found that the Okayama surveillance protocol was more intense, and we speculated that referring doctors might be more aware of HCC because the incidence was higher in the East.^28^–^31^

The effect of the differences in timing also translates into the RFS. The median RFS was 2 years in the Rotterdam cohort and 4 years and Okayama cohort, whereas the median RFS for the Hong Kong derivation cohort was even longer (5.5 years). This sizeable difference was observed in all other validation cohorts published by Chan et al.^5^ and also raises questions about the patient selection in the Hong Kong derivation cohort.^5^ The authors have not mentioned this result or investigated its origin. The impact on the predicted survival probabilities remains unclear. Although the survival data were censored at 24 months, the excellent long-term survival likely affected the baseline survival function. Because the baseline survival function is key in forming the predictions, it therefore also affects the accuracy of the prediction model.

Finally, it is important to note that our study had several limitations. First, the analysis was performed on validation cohorts with limited sample sizes. Especially conclusions for the high-risk group, clinically the most relevant, might have been unstable. Second, we recognize that the mechanisms for missing data might have differed across cohorts and that the complete case setup results in biased estimates if the data are not missing completely at random. However, following the derivation paper, we decided not to use multiple imputation techniques.

Although outside the scope of our research, model extension is needed to explain the differences in discriminatory power between Eastern and Western cohorts because they are clearly distinct. Also, we did not investigate the adequacy of the non-parametric baseline hazard. Parametric baseline hazard functions may improve the efficiency of the model.^32^ Additionally, stratification of the baseline hazard, dynamic covariates, and time-varying coefficients might prove to be fertile ground for improving the model. Finally, future research should focus on the implementation of prediction models into clinical decision-making. Arbitrary risk groups or abstract survival probabilities might prove to be hard for patients and doctors to incorporate intuitively into their decisions. Currently, a framework about how to translate predictions into care is lacking.

## Conclusions

In summary, this study showed that the discrimination of ERASL models may be poorer for Western patients than for Japanese patients, who showed good (or better) performance. The ERASL models require recalibration before risk prediction for individuals. We conclude that a new model needs to be developed that explains the East–West difference or is representative for Western patients.

## Supplementary Information

Below is the link to the electronic supplementary material.

Supplementary file1 (DOCX 603 kb)

Supplementary file1 (DOCX 45 kb)
