# Supplementary material for: Prediction of Early Recurrence After Surgery for Liver Tumor (ERASL): An International Validation of the ERASL Risk Models
Source: Ann Surg Oncol. 2021 Jul 7;28(13):8211–20. doi: 10.1245/s10434-021-10235-3 (PMC8591001; doi:10.1245/s10434-021-10235-3)
Supplement: Supplementary file 1 — Supplementary file1 (DOCX 603 kb) [file 10434_2021_10235_MOESM1_ESM.docx]

# Prediction of Early Recurrence After Surgery for Liver Tumor (ERASL): An International Validation of the ERASL Risk Models – Electronic supplements

Berend R Beumer BSc^1^; Kosei Takagi PhD^2^; Bastiaan Vervoort MsC^1^; Stefan Buettner PhD^1^; Yuzo Umeda PhD^2^; Takahito Yagi PhD^2^; Toshiyoshi Fujiwara PhD^2^; Ewout W Steyerberg PhD^3^; Jan NM IJzermans PhD^1^

Affiliations:

1 Department of Surgery, Division of HPB & Transplant Surgery, Erasmus MC, University Medical Centre

Rotterdam, Rotterdam, The Netherlands Erasmus MC Rotterdam

2 Department of Gastroenterological Surgery, Okayama University Graduate School of Medicine,

Dentistry, and Pharmaceutical Sciences, Okayama, Japan Okayama University Hospital

3 Department of Biomedical Data Science, Leiden University Medical Centre, Leiden, the Netherlands

Corresponding author and contact for reprints:

Prof. Dr. J.N.M. IJzermans,

Phone: 010-7032396

Email: [j.ijzermans@erasmusmc.nl](mailto:j.ijzermans@erasmusmc.nl)

**Electronic supplement 1**

Specification ERASL scores (1)

ERASL-pre score = 0.818 * Gender (0: Female, 1: Male)

+ 0.447 * ALBI grade (0: Grade 1; 1: Grade 2 or 3)

+ 0.100 * ln(Serum AFP in µg/L)

+ 0.580 * ln(Tumour size in cm)

+ 0.492 * Tumour number (0: Single; 1: Two or three; 2: Four or more)

Risk groups were assigned based on the following cut-offs: ≤2.558 (low), >2.558 to ≤3.521 (intermediate), and >3.521 (high).

ERASL-post score = 0.677 * Gender (0: Female, 1: Male)

+ 0.458 * ALBI grade (0: Grade 1; 1: Grade 2 or 3)

+ 0.082 * ln(Serum AFP in µg/L)

+ 0.451 * ln(Tumour size in cm)

+ 0.379 * Tumour number (0: Single; 1: Two or three; 2: Four or more)

+ 0.661 * Microvascular invasion (0: no, 1: yes)

Risk groups were assigned based on the following cut-offs: ≤2.332 (low), >2.332 to ≤3.445 (intermediate), and >3.445 (high).

Specification ALBI grade (1,2)

ALBI score = -0.085 * (albumin g/l) + 0.66 * log_10_(bilirubin µmol/l})

ALBI grade = 1 if ALBI score ≤ -2.60

1. if ALBI score > -2.60, ≤ -1.39
2. if ALBI score > -1.39

Abbreviations - AFP: alpha-fetoprotein µg/L, ALBI: albumin-bilirubin score

**References:**

1. Chan AW, Zhong J, Berhane S, Toyoda H, Cucchetti A, Shi K, et al. Development of pre and post-operative models to predict early recurrence of hepatocellular carcinoma after surgical resection. Journal of hepatology. 2018;69(6):1284-93.
2. Johnson PJ, Berhane S, Kagebayashi C, Satomura S, Teng M, Reeves HL, et al. Assessment of liver function in patients with hepatocellular carcinoma: a new evidence-based approach—the ALBI grade. Journal of Clinical Oncology. 2015;33(6):550.

**Electronic supplementary Figure 1: Survival function Median ERASL**


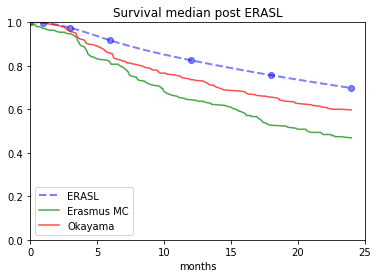

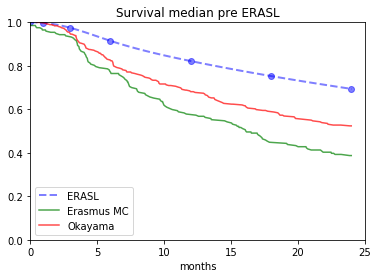


*Footnote Supplemental figure* *1.* The baseline survival curves at the median ERASL model are displayed for the Rotterdam (green) and Okayama (red) cohort. The baseline survival curves were scaled with the published median ERASL values. The blue dotted line is a cubic spline interpolation based on the values published by Chan et al.

**Electronic supplementary Figure 2: Calibration plot Okayama**


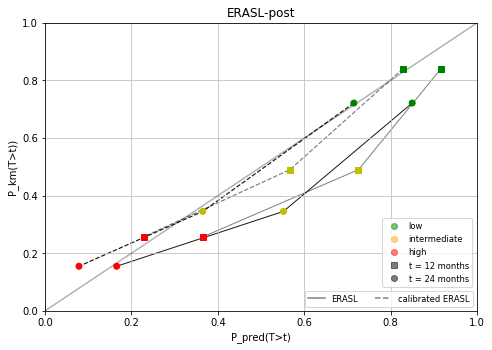

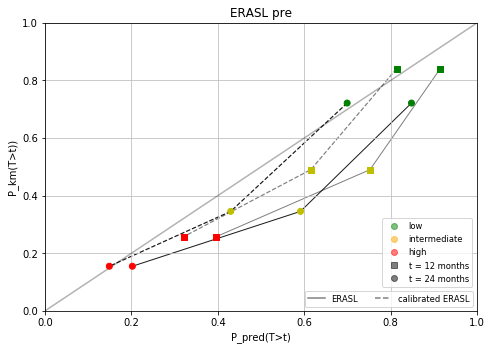


*Footnote Supplemental figure 2:* The calibration plot that displays predicted versus observed survival probabilities. The colours green, yellow, and red represent the various risk groups. The original and calibrated models are again distinguished by solid and dashed lines.

**Electronic supplementary Table 1**

**
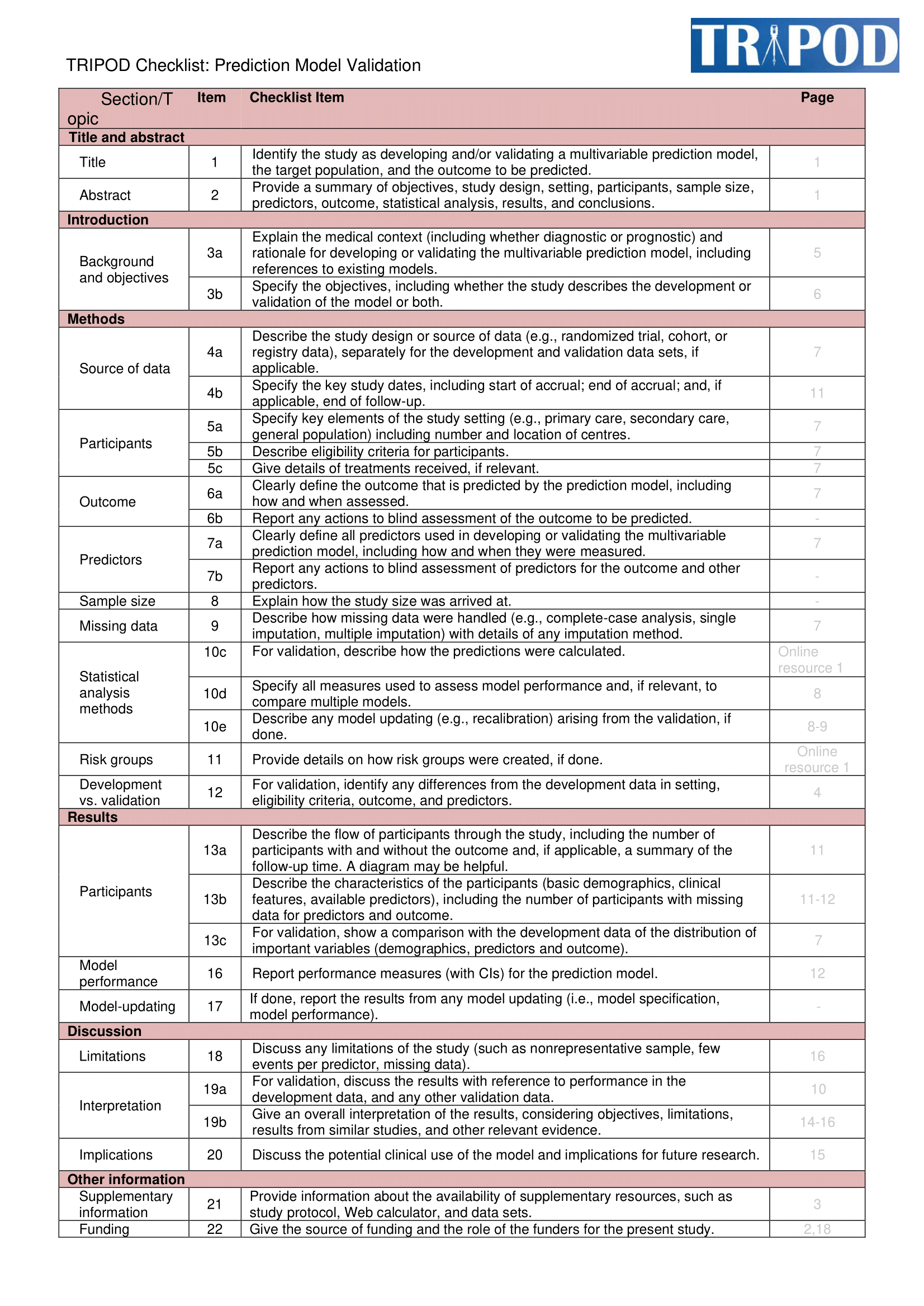
**

**Electronic supplementary Table 2: Hepatectomy subtypes**

| Resection category | Hepatectomy subtypes | Rotterdam n (%) | Okayama n (%) | Resected segments |
| --- | --- | --- | --- | --- |
| Major resection | Trisectionectomy Right * | 24 (9) | 3 (1) | 6+7+5+8+4 (+1) |
|  | Trisectionectomy Left * | 1 (0) | 3 (1) | 5+8+4+2+3 (+1) |
|  | Hemihepatectomy Right | 77 (28) | 69 (18) | 6+7+5+8 (+1) |
|  | Hemihepatectomy Left | 33 (12) | 59 (15) | 4+2+3 (+1) |
|  | Central bisectionectomy | **-** | 11 (3) | 5+8+4 |
| Minor resection | Segmentectomy ** | 103 (37) | 52 (13) | 1,2,3,5,6,7,8 |
|  | Sectionectomy ** | - | 147 (38) | 6+7, 5+8, 4, 2+3 |
|  | Wedge | 41 (15) | 48 (12) | Partial segment |

*Footnote Electronic supplementary Table2:* * For the Rotterdam cohort patients were registered as trisectionectomy also if only one additional segment to the hemi hepatectomy was resected. ** For Rotterdam sectionectomy was registered as segmentectomy. Abbreviations – n: number of patients.

**Electronic supplementary Table 3: Calibration slopes**

|  | ERASL-pre | | ERASL-post | |
| --- | --- | --- | --- | --- |
|  | Rotterdam | Okayama | Rotterdam | Okayama |
| Slope [95%CI] | 0.32 [0.04; 0.59] | 0.72 [0.54; 0.90] | 0.60 [0.33; 0.87] | 0.83 [0.65; 1.0] |
| p-value | <0.001 | 0.001 | 0.005 | 0.068 |

*Footnote Electronic supplementary Table 3:* Results of the Likelihood Ratio test to investigate if slope is significantly different from one. Abbreviations – CI: confidence interval.

**Electronic supplementary Table 4: Re-estimation regression ERASL-pre**

| **ERASL-pre** | Rotterdam | | | | Okayama | | | | | |
| --- | --- | --- | --- | --- | --- | --- | --- | --- | --- | --- |
|  | coef | exp(coef) | SE | p | coef | | exp(coef) | SE | p | |
| Gender | -0.78 | 0.46 | 0.20 | <0.001 | -0.83 | | 0.44 | 0.22 | <0.001 | |
| ALBI grade >1 | -0.49 | 0.61 | 0.24 | 0.041 | -0.10 | | 0.91 | 0.18 | 0.579 | |
| ln(AFP) | -0.01 | 0.99 | 0.03 | 0.618 | -0.05 | | 0.96 | 0.03 | 0.126 | |
| ln(Tumor size) | -0.43 | 0.65 | 0.14 | 0.002 | -0.14 | | 0.87 | 0.14 | 0.307 | |
| Tumor number | -0.41 | 0.66 | 0.20 | 0.034 | 0.06 | | 1.07 | 0.12 | 0.587 | |
| LR test with 5 df | 29.36 (p=<0.001) | | | | 21.6 (p=<0.001) | | | | | |
|  |  | | | |  | | | | | |
| **ERASL-post** | Rotterdam | | | | Okayama | | | | | |
|  | coef | exp(coef) | SE | p | coef | exp(coef) | | SE | | p |
| Gender | -0.66 | 0.52 | 0.21 | 0.002 | -0.63 | 0.53 | | 0.22 | | 0.004 |
| ALBI grade >1 | -0.63 | 0.53 | 0.26 | 0.015 | -0.00 | 1.00 | | 0.18 | | 0.980 |
| ln(AFP) | -0.01 | 0.99 | 0.03 | 0.806 | -0.03 | 0.97 | | 0.03 | | 0.276 |
| ln(Tumor size) | -0.28 | 0.76 | 0.15 | 0.058 | -0.29 | 0.75 | | 0.15 | | 0.055 |
| Tumor number | -0.30 | 0.74 | 0.20 | 0.138 | 0.17 | 1.19 | | 0.11 | | 0.131 |
| Microvascular- invasion | 0.16 | 1.18 | 0.22 | 0.046 | 0.25 | 1.29 | | 0.20 | | 0.206 |
| LR test with 6 df | 20.0 (p=0.003) | | | | 17.6 (p=0.007) | | | | | |

*Footnote Electronic supplementary Table 4:* The coefficients resulting from the offset regressions represent the difference between the coefficients published by Chan et al. (2018) and the coefficients obtained if the model would have been re-estimated on the validation data. Abbreviations - AFP: alpha-fetoprotein µg/L, ALBI: albumin-bilirubin score; Coef: coefficient; SE: standard error of the coefficient; p: p-value; LR: Likelihood Ratio; df: degrees of freedom.

**Electronic supplementary Table 5: Weibull calibration model**

|  | ERASL-pre | | | | ERASL-post | | | |
| --- | --- | --- | --- | --- | --- | --- | --- | --- |
|  | Rotterdam | | Okayama | | Rotterdam | | Okayama | |
|  | Coef (SE) | p | Coef (SE) | p | Coef (SE) | p | Coef (SE) | p |
| μ | -2.21 (0.41) | 0.00 | -1.05 (0.31) | 0.00 | -1.50 (0.40) | 0.00 | -0.83 (0.29) | 0.00 |
| γ | -0.39 (0.18) | 0.03 | -0.80 (0.11) | 0.00 | -0.67 (0.17) | 0.00 | -0.90 (0.11) | 0.00 |
| σ | 1.26 (0.09) | 0.01 | 1.11 (0.08) | 0.18 | 1.13 (0.09) | 0.16 | 1.07 (0.07) | 0.34 |

*Footnote Electronic supplementary Table 5: Results of the Weibull calibration model with* μ describing the modifications to the overall risk level, γ the impact of the linear predictor and controlling the shape of the baseline hazard function. Abbreviations – p: p-value, Coef: coefficient, SE: standard error.

**Electronic supplementary Table 6: Model extension**

|  | ERASL-pre | | | | ERASL-post | | | |
| --- | --- | --- | --- | --- | --- | --- | --- | --- |
|  | Rotterdam | | Okayama | | Rotterdam | | Okayama | |
|  | Coef [95%CI] | p | Coef [95%CI] | p | Coef [95%CI] | p | Coef [95%CI] | p |
| HBV | 0.39 [-0.02; 0.80] | 0.07 | 0.29 [-0.09; 0.67] | 0.13 | 0.42 [0.0; 0.84] | 0.06 | 0.24 [-0.13; 0.63] | 0.21 |
| HCV | 0.07 [-0.47; 0.62] | 0.79 | 0.27[-0.07; 0.60] | 0.12 | -0.08 [-0.65; 0.47] | 0.76 | 0.24 [-0.09; 0.58] | 0.16 |

*Footnote Electronic supplementary Table 6:* Results of extending the ERASL models with the variables HBC and HCV. Abbreviations – Coef: coefficient, CI: confidence interval, p: p-value, HBV: hepatitis B virus, HCV: hepatitis C virus.

**Electronic supplementary Table 7: Forward selection Rotterdam**

*A: Rotterdam ERASL-pre*

|  | round 1: ERASL-pre | |  | round2: ERASL-pre + Ln(AFP) | | |
| --- | --- | --- | --- | --- | --- | --- |
|  | Coef [95%CI] | p |  | Coef [95%CI] | p | |
| Gender | -0.34 [-0.77; 0.09] | 0.12 |  | -0.14 [-0.62; 0.34] | | 0.58 |
| ALBI grade >1 | -0.22 [-0.73; 0.29] | 0.39 |  | -0.12 [-0.63; 0.39] | | 0.64 |
| Ln(AFP) | **0.08 [0.02; 0.14]** | **0.02** |  |  | |  |
| Ln(Tumor size) | 0.04 [-0.27; 0.35] | 0.81 |  | 0.12 [-0.20; 0.44] | | 0.44 |
| Tumor number | -0.09 [-0.49; 0.31] | 0.68 |  | -0.01 [-0.42; 0.40] | | 0.96 |
| HBV | 0.20 [-0.22; 0.62] | 0.34 |  | 0.10 [-0.33; 0.53] | | 0.63 |
| HCV | 0.11 [-0.43; 0.65] | 0.69 |  | 0.08 [-0.46; 0.62] | | 0.76 |

*B: Rotterdam ERASL-post*

|  | round 1: ERASL-post | | round 2: ERASL-post + microvascular | | round 3:  ERASL-post + microvascular + Ln(AFP) | |
| --- | --- | --- | --- | --- | --- | --- |
|  | Coef [95%CI] | p | Coef [95%CI] | p | Coef [95%CI] | p |
| Gender | -0.45 [-0.89; -0.01] | 0.05 | -0.35 [-0.8; 0.1] | 0.13 | -0.14 [-0.65; 0.37] | 0.59 |
| ALBI grade >1 | -0.51 [-1.04; 0.02] | 0.06 | -0.39 [-0.94; 0.16] | 0.04 | -0.26 [-0.82; 0.3] | 0.35 |
| Ln(AFP) | 0.05 [-0.01; 0.11] | 0.09 | **0.07 [0.01; 0.13]** | ***0.03*** |  |  |
| Ln(Tumor size) | -0.03 [-0.34; 0.28] | 0.86 | 0.08 [-0.24; 0.4] | 0.62 | 0.17 [-0.16; 0.5] | 0.32 |
| Tumor number | -0.09 [-0.49; 0.31] | 0.66 | -0.04 [-0.44; 0.36] | 0.84 | 0.03 [-0.38; 0.44] | 0.87 |
| microvascular | **0.64 [0.12; 1.16]** | **0.02** |  |  |  |  |
| HBV | 0.35 [-0.07; 0.77] | 0.11 | 0.3 [-0.12; 0.72] | 0.16 | 0.22 [-0.21; 0.65] | 0.33 |
| HCV | 0.00 [-0.56; 0.56] | 0.99 | -0.06 [-0.63; 0.51] | 0.84 | -0.07 [-0.63; 0.49] | 0.80 |

*Footnote Electronic supplementary Table 7:* Results of forward selection the ERASL models in the Rotterdam cohort. Abbreviations – Coef: coefficient, CI: confidence interval, p: p-value, ALBI: albumin-bilirubin score, AFP: alpha-fetoprotein µg/L, HBV: hepatitis B virus, HCV: hepatitis C virus.

**Electronic supplementary Table 8: Forward selection Okayama**

*A: Okayama ERASL-pre*

|  | round 1: ERASL-pre | |  | round 2: ERASL-pre + Gender | |
| --- | --- | --- | --- | --- | --- |
|  | Coef [95%CI] | p |  | Coef [95%CI] | p |
| Gender | **-0.67 [-1.12; -0.22]** | ***0.00*** |  |  |  |
| ALBI grade >1 | 0.03 [-0.35; 0.41] | 0.88 |  | 0.01 [-0.37; 0.39] | 0.97 |
| Ln(AFP) | 0.00 [-0.07; 0.07] | 0.95 |  | -0.04 [-0.11; 0.03] | 0.26 |
| Ln(Tumor size) | 0.05 [-0.31; 0.41] | 0.80 |  | -0.11 [-0.49; 0.27] | 0.58 |
| Tumor number | 0.33 [0.05; 0.61] | 0.02 |  | 0.22 [-0.08; 0.52] | 0.14 |
| HBV | 0.20 [-0.19; 0.59] | 0.32 |  | 0.24 [-0.15; 0.63] | 0.23 |
| HCV | 0.11 [-0.23; 0.45] | 0.52 |  | 0.11 [-0.24; 0.46] | 0.55 |

*B: Okayama ERASL-post*

|  | round 1: ERASL-post | |  | round 1: ERASL-post + Gender | |
| --- | --- | --- | --- | --- | --- |
|  | Coef [95%CI] | p |  | Coef [95%CI] | p |
| Gender | **-0.62 [-1.06; -0.18]** | **0.01** |  |  |  |
| ALBI grade >1 | 0.00 [-0.37; 0.37] | 0.99 |  | 0.00 [-0.37; 0.37] | 0.99 |
| Ln(AFP) | -0.01 [-0.07; 0.05] | 0.68 |  | -0.04 [-0.1; 0.02] | 0.18 |
| Ln(Tumor size) | -0.25 [-0.63; 0.13] | 0.2 |  | -0.38 [-0.77; 0.01] | 0.06 |
| Tumor number | 0.29 [0.04; 0.54] | 0.03 |  | 0.22 [-0.04; 0.48] | 0.09 |
| microvascular | 0.37 [-0.07; 0.81] | 0.1 |  | 0.23 [-0.22; 0.68] | 0.32 |
| HBV | 0.21 [-0.18; 0.6] | 0.29 |  | 0.24 [-0.15; 0.63] | 0.22 |
| HCV | 0.17 [-0.17; 0.51] | 0.33 |  | 0.17 [-0.18; 0.52] | 0.34 |

*Footnote Electronic supplementary Table 8:* Results of forward selection the ERASL models in the Okayama cohort. Abbreviations – Coef: coefficient, CI: confidence interval, p: p-value, ALBI: albumin-bilirubin score, AFP: alpha-fetoprotein µg/L, HBV: hepatitis B virus, HCV: hepatitis C virus.
